# Extension of Weighted Sum of Gray Gas Data to Mathematical Simulation of Radiative Heat Transfer in a Boiler with Gas-Soot Media

**DOI:** 10.1155/2014/504601

**Published:** 2014-03-06

**Authors:** Samira Gharehkhani, Ali Nouri-Borujerdi, Salim Newaz Kazi, Hooman Yarmand

**Affiliations:** ^1^Department of Mechanical Engineering, University of Malaya, 50603 Kuala Lumpur, Malaysia; ^2^School of Mechanical Engineering, Sharif University of Technology, Tehran 11365-956, Iran

## Abstract

In this study an expression for soot absorption coefficient is introduced to extend the weighted-sum-of-gray gases data to the furnace medium containing gas-soot mixture in a utility boiler 150 MWe. Heat transfer and temperature distribution of walls and within the furnace space are predicted by zone method technique. Analyses have been done considering both cases of presence and absence of soot particles at 100% load. To validate the proposed soot absorption coefficient, the expression is coupled with the Taylor and Foster's data as well as Truelove's data for CO_2_-H_2_O mixture and the total emissivities are calculated and compared with the Truelove's parameters for 3-term and 4-term gray gases plus two soot absorption coefficients. In addition, some experiments were conducted at 100% and 75% loads to measure furnace exit gas temperature as well as the rate of steam production. The predicted results show good agreement with the measured data at the power plant site.

## 1. Introduction

One of the most important modes of heat transfer in a boiler furnace of a large power plant is radiation. Thus determination of the radiative properties of combustion products is vital to predict the temperature and heat flux distribution. The main products of combustion in an enclosure contain carbon dioxide and water vapor [1]. Some studies have been done on modeling of gas mixture in furnaces. Different models for calculation of the radiative properties of real gases have been proposed by researchers such as statistical narrow band (SNB), the full-spectrum correlated-k (FSCK) distribution, and the weighted-sum-of-gray gases (WSGG) [2–6]. Among these models, the last one is more reasonable in engineering calculations in view of the accuracy and computing time. The WSGG model was developed by Hottel and Sarofim [1]. Modest [7] stated that this model can be applied for any solution methods for the transport equation. Based on WSGG concept, Taylor and Foster [8] utilized a “three gray plus one clear gas” model. A three-term mixed gray gas model with third order polynomial for weighting factors was employed by Smith et al. [9]. Soufiani and Djavdan [10] proposed a five-order polynomial for gas combustion, where *P*_*w*_/*P*_*c*_ = 2. After that a lot of efforts were given to model the radiative properties from semitransparent media containing a mixture of nongray gases and soot [11, 12] which was applied for analyzing the radiative heat transfer inside the furnace [13]. Among the different methods of modeling the combusting environments such as inverse methodologies, hybrid method, discrete-ordinates method, and finite volume method [14–17], the zone method is more practical and many attempts on radiative heat transfer analysis were conducted based on this method [18–20]. Zone method was originally developed by Hottel and Cohen [21] for an absorbing, emitting, nonscattering gray gas with constant absorption coefficient. Later Hottel and Sarofim [1] extended it to deal with three-dimensional problems. Also Larsen and Howell [22] presented a method for calculations related to the direct exchange areas in zonal analysis based on last-squares smoothing. Tucker [23] conducted a numerical integration and suggested an exponential expression for exchange areas which covers a range of optical thickness from 0 to 18. Lawson [24] proposed an improved method for smoothing approximate exchange areas. To achieve the total exchange areas, Noble [25] presented the explicit matrix relations. Batu and Selçuk [26] analyzed the radiative heat transfer in the freeboard of a fluidized bed combustor by using the zone method. Bordbar and Hyppanen [27] employed the zone method for predicting temperature and heat flux on the water walls of a steam boiler furnace. Recently, Méchi and coworkers [19] proposed a radiative model to extend the zonal method to semitransparent inhomogeneous composed of nongray gas and soot. Also Moghari et al. [18] used the zone method to predict thermal radiation behavior in the D-type water-cooled steam boiler furnace. Crnomarkovic et al. [28] used the simple gray gas (SGG) and WSGG to model the radiative properties of the two-phase mixture composed of gas and particles inside the lignite fired furnace.

In this study a new expression for soot absorption coefficient has been presented depending on temperature which could be coupled with nonluminous flame data containing several gray gases and one clear gas. The results are based on the suggested soot absorption coefficient coupled with the data generated by Taylor and Foster. The validity of the calculated soot absorption coefficient is confirmed by comparison with the obtained total emissivities and the calculated values from Truelove's models. In addition, for reconfirmation of the results, the soot expression is utilized in the zone method to model the furnace of a utility boiler 150 MWe. The temperature and heat flux distributions are discussed for 2 cases (with and without soot particles) at 100% load. Furthermore the furnace exit gas temperature and amount of steam production by considering the effect of soot for the loads of 100%; 75% are presented and compared with the captured data from the site.

## 2. Mathematical Model

### 2.1. The Weighted Sum of Gray Gas

The weighted-sum-of-gray gases (WSGG) is one of the accurate techniques for modeling the radiative behavior of combustion gases. The total emissivity of real gas can be represented mathematically by a mixture of *N* gray gases [1]:

(1) $$\begin{array}{c} \varepsilon_{g}=\sum_{n=1}^{N}a_{g,n}\left[1-\exp\left(-K_{g,n}\left(P\right)L\right)\right], \end{array}$$

where  *K*_*g*,*n*_, *P*, and *L* represent the absorption coefficient for the *n*th gray gas, sum of the partial pressure of all radiating gases in the mixture, and effective path length, respectively, and  *a*_*g*,*i*_  is weighting factors [5, 9, 10, 12] of various commonly used correlations for the mixture of combustion products which have been reported by Taylor and Foster [8], Smith et al. [9], and Soufiani and Djavdan [10].

In fact WSGG is an appropriate tool which could be applied in the modeling of media containing CO_2_, H_2_O, and soot and, in this subject, some approaches have been developed to consider the effect of soot particles [12, 29].

Based on the suggestion of Truelove [12] for the gas-soot mixture, the two absorption coefficients (gas mixture and soot) are contributed in the calculations. The expressions for emissivity of the combustion product-soot mixture  (*ε*_*m*_)  can be presented by:

(2) $$\begin{array}{c} \varepsilon_{m}=\sum_{n=1}^{N}a_{n\;}\left(T\right)\left[1-\exp\left\{-K_{g,n}\left(P\right)L-K_{s}C_{s}L\right\}\right], \end{array}$$

where  *C*_*s*_ is the soot concentration.

In order to determine the soot absorption coefficient, a relationship from the wavelength dependence of  *K*_*s*,*λ*_ derived from experimental investigations is [30] as follows:

(3) $$\begin{array}{c} K_{s,\lambda }=a\lambda^{-b}, \end{array}$$

where  *a* = 2.71 × 10^3^ and  *b* = 1.090.

By integrating  *K*_*s*,*λ*_  over wavelength we have

(4) $$\begin{array}{c} K_{s}=\frac{1}{\sigma T^{4}}\int_{0}^{\infty }K_{s,\lambda }e_{b,\lambda }d\lambda =\frac{1}{\sigma T^{4}}\int_{0}^{\infty }\frac{2\pi C_{1}a\lambda^{-b}d\lambda }{\lambda^{5}\left(e^{\left(C_{2}/\lambda T\right)}-1\right)}. \end{array}$$

Introducing  *z* = *C*_2_/*Tλ*  into above equation, the soot absorption coefficient becomes

(5) Ks=2πaC1(T/C2)4+bσT4∫0∞z3+b  (ez−1)dz=2πaC1(T/C2)4+bσT4Γ(4+b)ξ(4+b),

where *C*_1_  = 3.742 × 10 W·*μ*m^4^/m^2^ and *C*_2_  = 1.4388 × 10^4^ *μ*m·K are the first and second Planck function constants, respectively, *σ*  = 5.669 × 10^8^ W m^−2 ^K^−4^ is Stephane-Boltzman constant,  Γ(*z*)  is Gamma function, and *ξ*(*z*)  is Rieman zeta function. The above temperature dependence  *K*_*s*_ as determined from (5) is expressed by following the simple polynomial equation:

(6) $$\begin{array}{c} K_{s}=-43.37+0.6691T+2\times 10^{-5}T^{2}, \end{array}$$

where *T* is the temperature of the radiation source in Kelvin.

### 2.2. Zonal Method

In zone method the enclosure is subdivided into surfaces and volumes zones which could be assumed isothermal [31]. Then by using the gas flow and combustion pattern, the mass flow rate from/to each volume zone, generated heat by combustion and convection coefficients are obtained. A steady state energy balance is considered for each zone and then a set of simultaneous equations based on the temperatures and heat fluxes are produced. By solving these equations, the temperature and heat flux distributions are obtained.

**Figure 1:**
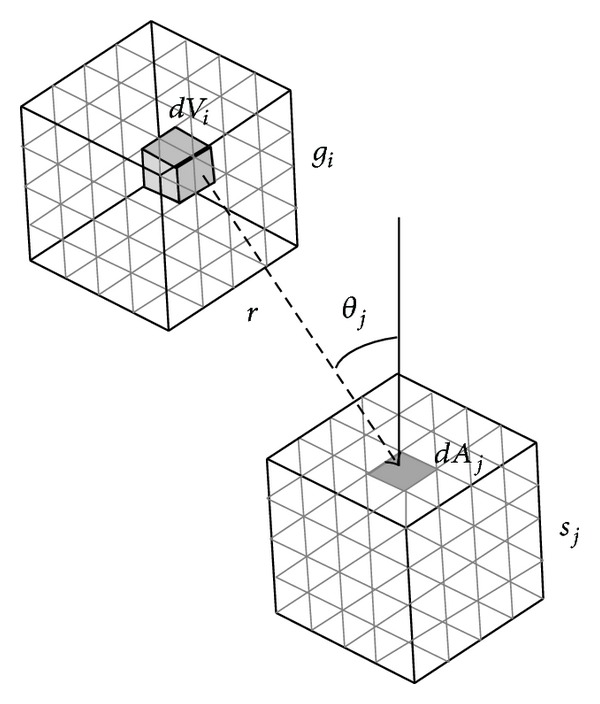
The schematic of volume and surface zone.

*Calculation of the Direct Exchange Areas and Total Exchange Areas*. For finding the radiative heat transfer between two zones, the first step is to calculate the direct exchange areas (DEA) and then the total exchange areas (TEA). There are three types of DEAs: surface-surface, volume-surface, and volume-volume. For instant, the volume-surface direct exchange area as illustrated in Figure 1 can be determined as follows:

(7) $$\begin{array}{c} \bar{g_{i}s_{j}}=\int_{V_{i}}\int_{A_{j}}\frac{K_{t}\cos\theta_{j}\exp\left(-K_{t}r_{ij}\right)}{\pi r_{ij}^{2}}dV_{i}dA_{j}. \end{array}$$

The DEAs obey the reciprocity definitions where  $\bar{s_{i}s_{j}}=\bar{s_{j}s_{i}}$ and  $\bar{g_{i}g_{j}}=\bar{g_{j}g_{i}}$. Direct numerical integration can be applied to calculate the respective areas.

For the gray gas the total flux between two zones *i* and *j* must be proportional to  *σ*(*T*_*i*_^4^ − *T*_*j*_^4^)  and the proportionality constant, called the total exchange area, is indicated by$\;\bar{SS},\bar{GS},\bar{GG}$ [1]. All of these terms are calculated by using the methods that have been reported by Hottel and Sarofim [1] and Modest [7].

*Direct Flux Areas*. The radiant energy between any two zones is proportional to the a-weighted summation of the total exchange areas for each gas. For example, the net flux between zones *i* and *j* is given by [1]:

(8) Q˙ij={∑n=1N[ag,n(Ti)](GiSj¯)n}Eg,i −{∑n=1N[as,n(Tj)](GiSj¯)n}×Es,j≡GiSj→Eg,i−GiSj←Es,j,

where$\;\vec{G_{i}S_{j}}\;$and$\;\overset{\leftarrow }{G_{i}S_{j}}\;$are replacing the terms in the brackets. These are called directed-flux areas [1, 29]. Similarly expression for surface-surface transfer is

(9) $$\begin{array}{c} Q_{ij}=\vec{S_{i}S_{j}}E_{s,i}-\overset{\leftarrow }{S_{i}S_{j}}E_{s,j}. \end{array}$$

And for gas-gas transfer it is expressed by

(10) $$\begin{array}{c} Q_{ij}=\vec{G_{i}G_{j}}E_{g,i}-\overset{\leftarrow }{G_{i}G_{j}}E_{g,j}. \end{array}$$

*Total Energy Balance*. For a volume zone *i*, the total energy balance can be stated by

(11) ∑j=1lGiGj←Eg,j+∑j=1mGiSj←Es,j  −4∑n=1N[ag,n(Tg)Kg,nViEg,i]−(Q˙conv)i  +(Q˙G,net+Q˙a)i+(Q˙enth)i=0,

where  *l*  and *m* are the number of volume and surface zones, respectively. *N* is the number of gases in the model, ${\left({\dot{Q}}_{\text{conv}}\right)}_{i}\;$is the convection heat transfer to all surfaces in contact with the volume zone, and$\;{\left({\dot{Q}}_{\text{enth}}\right)}_{i}\;$is the total sensible heat presented by

(12) $$\begin{array}{c} {\dot{Q}}_{\text{enth}}=m_{i^{\prime }\to i}{\left(C_{P}T\right)}_{i^{\prime }}-m_{ti}{\left(C_{P}T\right)}_{i}, \end{array}$$

where  *m*_*i*′→*i*_  is the mass flow rate of gas entering the zone *i* from a neighboring zone *i*′, and  *m*_*ti*_  is representing the total mass flow rate of gas leaving the zone *i*. Also ${\left({\dot{Q}}_{G,\text{net}}+{\dot{Q}}_{a}\right)}_{i}$ is heat released due to combustion plus the heat content in the combustion air, so this term can be expressed [27]:

(13) $$\begin{array}{c} {\dot{Q}}_{G,\text{net}}+{\dot{Q}}_{a}={\dot{V}}_{G}\left[C_{v,\text{net}}+R_{s}\left(1+\frac{X}{100}\right)\rho_{a}^{o}\left(H_{a}\left(T_{a}\right)\right)\right]. \end{array}$$

On the other hand for a surface zone *i*, the total energy balance could be represented by

(14) $$\begin{array}{c} \sum_{j=1}^{m}\overset{\leftarrow }{S_{i}S_{j}}E_{s,j}+\sum_{j=1}^{l}\vec{G_{j}S_{i}}E_{g,j}-A_{i}\varepsilon_{i}E_{s,i}+A_{i}{\dot{q}}_{i,\text{conv}}={\dot{Q}}_{i}, \end{array}$$

where ${\dot{Q}}_{i}$ is heat transfer rate to water walls.

Finally, the energy balance for the total number of volume and surface zones generates a series of nonlinear algebraic equations. These equations should be solved by the iterative techniques in order to achieve the temperature distribution in zones. In this study, the surfaces have been assumed gray and the combustion is complete in the zones in front of burners.

## 3. Experimental Facility

**Figure 2:**
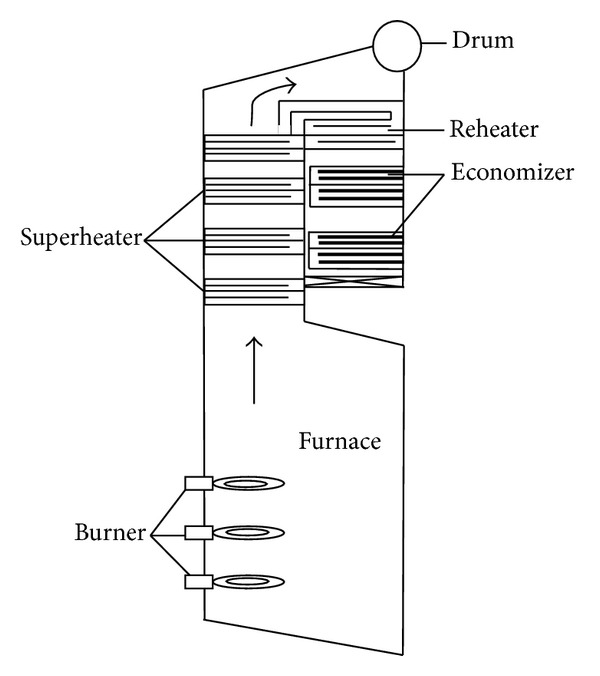
Overview of boiler.

The experimental data were obtained from the furnace of a 150 MWe utility boiler. Schematic of boiler is shown in Figure 2. The dimensions of the boiler furnace are  9.2 m × 9.2 m × 23 m  which is equipped with 9 natural gas fired burners in three rows of three. They are located at the left side wall of the furnace chamber. The operating conditions of the boiler and fuel characteristics are mentioned in Table 1. The experiments were conducted to measure the furnace exit gas temperature by a thermocouple with reasonable accuracy (0.05% of reading) located adequately far from the last raw of burners at the furnace outlet at 100% and 75% loads.

## 4. Results and Discussion

The soot absorption coefficient suggested in (6) can be coupled with available models for nonluminous flam with gray gases and one clear gas to obtain the total emissivities of gas-soot mixture and to be applied in the zone method.

**Figure 3:**
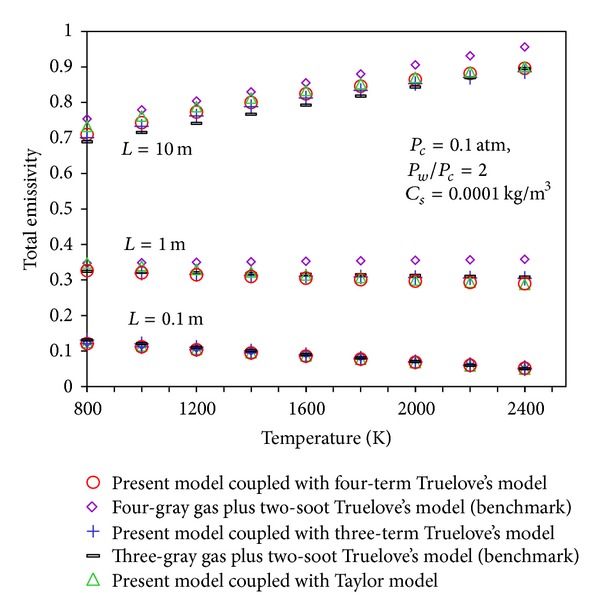
Total emissivity based on present calculations and Truelove's models for gas combustion, *P*_*w*_/*P*_*c*_ = 2.

*Validation of Presented Soot Absorption Coefficient with Truelove's Model.* The total emissivities of gas-soot mixture are obtained by coupling the calculated  *K*_*s*_ by using (6) and Taylor's data. The evaluated total emissivities at the different temperatures with soot concentration of 0.0001 Kg/m^3^ for gas combustion are well compared against benchmark data which are “three-gray gas plus two-soot” and “four-gray gas plus two-soot” models suggested by Truelove (Figure 3). Also to show the suitability of using the present expression in other models, the suggested  *K*_*s*_  is coupled with Truelove's models without soot which are three-term (two-gray plus one clear) and four-term (three-gray plus one clear) gas models and the results are presented in Figure 3 as well.

Table 2 presents the discrepancies between the computed total emissivities by using the coupled models and benchmarks.

**Figure 4:**
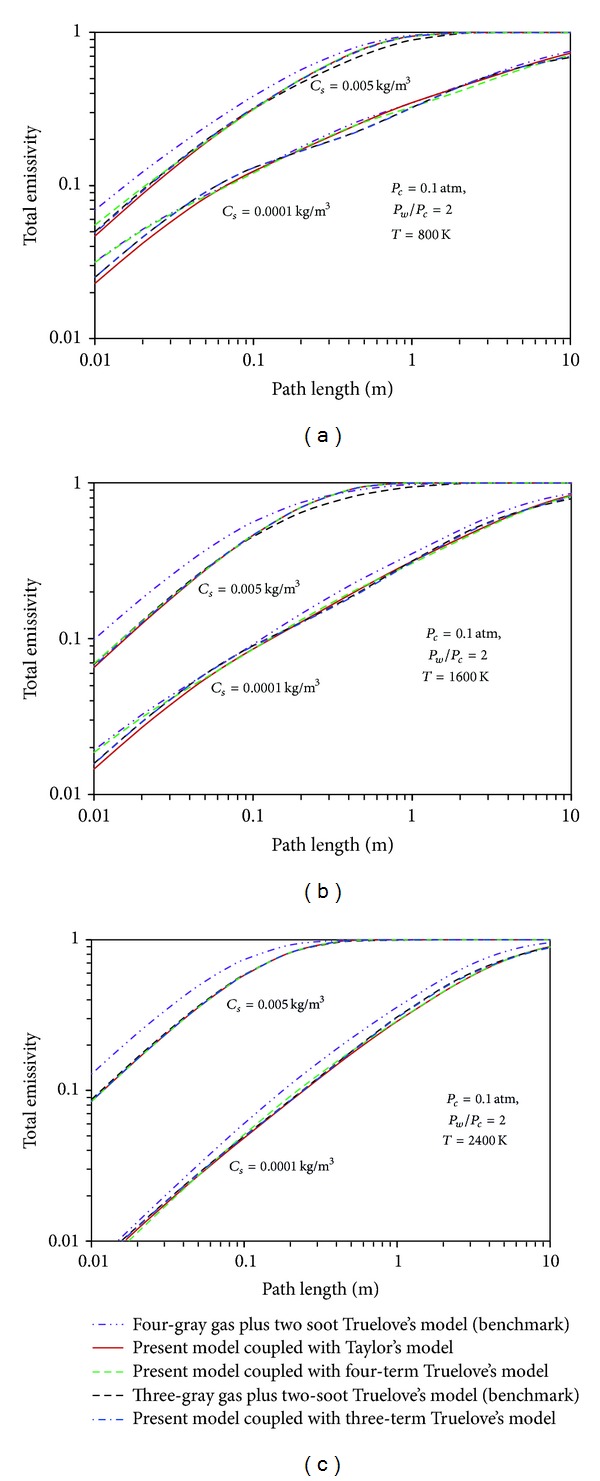
Total emissivity of the coupled models and benchmarks for different path lengths and soot concentrations.

Figure 4 demonstrates the comparison between the calculated gas-soot mixture total emissivity and benchmark data. The total emissivities are plotted versus path length on a logarithmic axis with two different soot concentrations (0.0001 Kg/m^3^ and 0.005 Kg/m^3^) for gas, at 800, 1600, and 2400°K.

It is seen that the obtained results coincide with the benchmarks; deviations are acceptable specially in comparison of two cases of models: (i) present model coupled with Taylor's data and (ii) present model coupled with 3-term Truelove's data with the Truelove's model (three-gray gas plus 2-soot). The errors are not greater than 4.8 percent at *L* > 0.1 m for the first case and 2 percent at all path lengths for second case for small soot concentration (0.0001 Kg/m^3^) at 1600°K.

**Figure 5:**
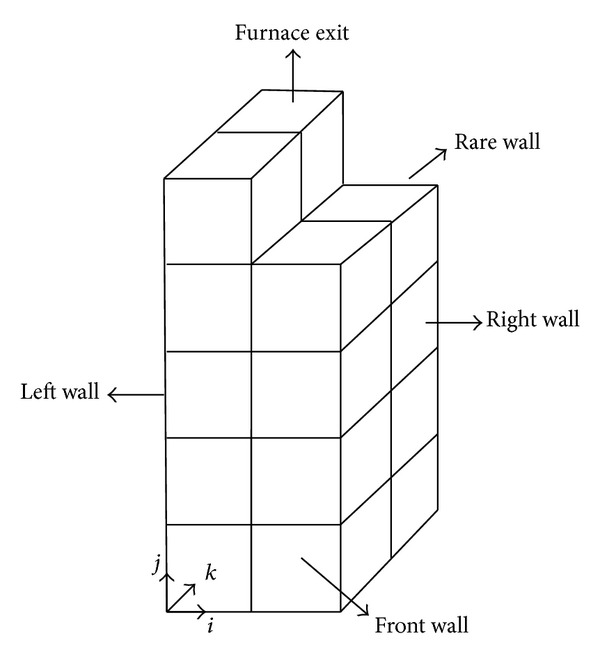
Schematic of the simplified model of the furnace.

*Application in the Zone Method*. The proposed soot absorption coefficient coupled with Taylor's data is used for radiative heat transfer analysis inside the boiler furnace. The emissivity of tube wall is considered 0.8. The zones of furnace are obtained by dividing the height (*j* direction) into five equal sections, the length (*i* direction) into 2 equal sections, and the width (*k* direction) into 2 equal sections; thus the furnace has been divided into 46 surface and 18 volume zones, as shown in Figure 5.

The results are based on the effects of existence and absence of soot particles. Tables 3 and 4 show the temperature distribution on height direction of front wall at 100% load for case 1 (excluding soot particles) and case 2 (including soot particles). It is noticeable that in the results of case 2, the *K*_*s*_ value is fixed at an average of zones temperature which is obtained from results by excluding soot particles. The amount of excess air is 5% and soot concentration is 0.00005 Kg/m^3^.

**Figure 6:**
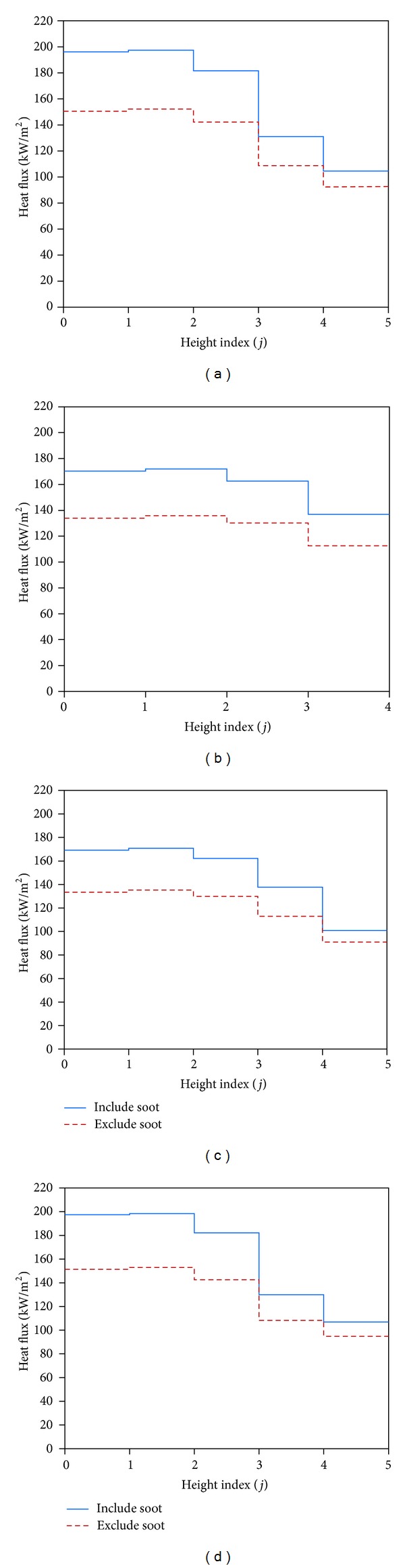
Heat flux distribution with and without soot particles along the (a) front wall (*i* = 1, *j*), (b) front wall (*i* = 2, *j*), (c) right wall (*k* = 1, *j*), and (d) left wall (*k* = 1, *j*).

The heat flux distribution along walls (front wall, right side wall, and left side wall) for both cases with soot and without soot is shown in Figure 6. It is observed that existence of soot raised considerably the heat flux on the wall. Similar result was reported in the literature [19, 32–34]. In fact the existence of soot enhances the radiative intensity because of continuum radiation in the visible and infrared regions of the wavelength spectrum [35] and in this situation the radiative transfer is conducted towards the wall and as a result of this, temperature of the medium has reduced.

To confirm the obtained results and validate the applied mathematical model, Tables 5 and 6 present a comparison between the calculated data and measured practical data. Available results of measurements are furnace exit gas temperature and rate of steam production at 100% and 75% loads.

The table shows that there is a good agreement between the present results and the experimental data.

## 5. Conclusions

A new soot absorption coefficient proposed in this paper has been assessed though coupling with WSGG parameters suggested by Taylor. It has been utilized for modeling the radiation heat transfer in a utility boiler 150 MWe. The total emissivities are calculated and compared with the Truelove's parameters for 3-term and 4-term gray gases plus two-soot absorption coefficients. In addition, some experiments were conducted at 100% and 75% loads to measure furnace exit gas temperature as well as the rate of steam production and the following results are obtained.

1. The soot absorption coefficient model is compatible with WSGG models containing gray gases and one clear gas.
2. The existence of soot particles leads to a decrease in gas temperature and an increase in wall heat flux.
3. The exhaust gas temperature and steam production could be estimated with reasonable accuracy at different loads.

## Figures and Tables

**Table 1:** Plant operation at 100% load and fuel characteristic.

Boiler load (MWe)	150
Fuel lower heating value (Kj/Kg)	50000
Fuel flow rate (Kg/hr)	30597
Fuel temperature °C	30
Excess air ratio %	5
Ambient temperature °C	28

Fuel chemical composition (%Vol)	
CH_4_	90.7
C_2_H_6_	6.2
C_3_H_8_	2.1
C_4_H_10_	1

**Table 2:** Comparison of the calculated total emissivities using the coupled models, with the benchmarks *T* = 1600°K and *C*_*s*_ = 0.0001 Kg/m^3^.

Present model coupled by:	Discrepancies (%) with “3-gas plus 2-soot” Truelove's model			Discrepancies (%) with “4-gas plus 2-soot” Truelove's model [12]		
	*L* = 0.1 m	*L* = 1 m	*L* = 10 m	*L* = 0.1 m	*L* = 1 m	*L* = 10 m
Taylor's data	−4.85	−0.679	5.25	−6.71	−10.88	−2.46
3-term Truelove's data	−0.768	−1.83	2.49	−2.70	−11.92	−5.02
4-term Truelove's data	−5.14	−3.61	−3.98	−6.99	−13.51	−3.64

**Table 3:** Temperature distribution in furnace with and without soot effect, *i* = 1 and *j* = 1,2,…, 5.

Height index, *j*	Temperature, K Case 1 (without soot)	Temperature, K Case 2 (with soot)
1	1796.0	1719.1
2	1803.4	1724.0
3	1781.8	1699.5
4	1643.2	1532.9
5	1587.5	1473.2

**Table 4:** Temperature distribution in furnace with and without soot effect, *i* = 2 and *j* = 1,2,…, 4.

Height index, *j*	Temperature, K Case 1 (without soot)	Temperature, K Case 2 (with soot)
1	1721.3	1628.4
2	1728.7	1633.5
3	1719.4	1622.0
4	1676.7	1571.7

**Table 5:** Comparison of the calculated furnace exit gas temperature with the measured data.

Load (%)	Gas temperature (K)		Discrepancy (%)
	Experimental data	Present data	
100	1605	1473.2	−8.2
75	1465	1396.9	−4.64

**Table 6:** Comparison of the calculated steam generation rates with measured data.

Load (%)	100	75
Fuel flow rate (Ton/h)	30.5	22.9
Measured in the site (Ton/h)	503	375
Calculated data (Ton/h)	492.5	401.58
Discrepancy (%)	−2.08	−6.9

## Nomenclature

*A*: Surface area (m^2^)
*a*: Weighting factor
*C*: Mass concentration (Kg/m^3^)
*C*_1_: First Planck function constant (W *μ*m^4^/m^2^)
*C*_2_: Second Planck function constant (*μ*m K)
*C*_*P*_: Specific heat capacity at constant pressure (kJ/kg K)
*E*: Black body emissive power (W/m^2^)
$\bar{g_{i}g_{j}},\bar{G_{i}G_{j}},\vec{G_{i}G_{j}}:$: Direct exchange area, total exchange area, and flux exchange area for volume *i* to volume *j*, (m^2^)
$\bar{g_{i}s_{j}},\bar{G_{i}s_{j}},\vec{G_{i}s_{j}}$: Direct exchange area, total exchange area, and flux exchange area for volume *i* to surface *j*, (m^2^)
*K*: Extinction coefficient of the medium (m^−1^)
*K*_*s*_: Soot absorption coefficient (m^2^/Kg)
*L*: Effective path length (m)
*N*: Number of gases
*P*: Partial pressure of gas (atm)
$\dot{Q}$: Heat transfer rate (W)
*R*: Distance between two zones (m)
*R*_*s*_: Stoichiometric air/fuel volume ratio
$\bar{s_{i}s_{j}},\bar{S_{i}S_{j}},\vec{S_{i}S_{j}}$: Direct exchange area, total exchange area, and flux exchange area for surface *i* to surface *j*, (m^2^)
*T*: Temperature (K)
*V*: Volume (m^3^)
*X*: Percentage excess air level.

### Greek Symbols

*α*: Gas absorptivity
*ε*: Gas emissivity
*ε*_*m*_: Emissivity of gas-soot mixture
*θ*: Angle between the beam joining the center points of two zones and the normal to one of the two zones (Rad)
Γ(*z*): Gamma function
*ξ*(*z*): Rieman zeta function
*σ*: Stephane Boltzman constant (W/m^2^ K^4^)
*ρ*: Density (Kg/m^3^).

### Subscripts

*a*: Air
*g*: Gas
*i*, *j*: Surface or volume zone
*l*: Number of volume
*m*: Number of surface
*n*: *n*th gray gas
*s*: Surface or soot.
